# A retrospective study on long-term efficacy of intranasal lysine-aspirin in controlling NSAID-exacerbated respiratory disease

**DOI:** 10.1007/s00405-021-07063-2

**Published:** 2021-09-04

**Authors:** Alfonso Luca Pendolino, Glenis K. Scadding, Bruno Scarpa, Peter J. Andrews

**Affiliations:** 1 Department of ENT, Royal National ENT & Eastman Dental Hospitals, 47-49 Huntley St, Bloomsbury, London, WC1E 6DG UK; 2 Ear Institute, University College London, London, UK; 3 Faculty of Medical Sciences, University College London, London, UK; 4 Department of Statistical Sciences and Department of Mathematics Tullio Levi-Civita, University of Padua, Padua, Italy

**Keywords:** Anti-inflammatory agents non-steroidal, Asthma, Aspirin-induced, Nasal polyps, Smell, Aspirin

## Abstract

**Purpose:**

Aspirin treatment after desensitization (ATAD) represents an effective therapeutic option suitable for NSAID-exacerbated respiratory disease (N-ERD) patients with recalcitrant disease. Intranasal administration of lysine-aspirin (LAS) has been suggested as a safer and faster route than oral ATAD but evidence for its use is less strong. We investigated nasal LAS therapy long-term efficacy based on objective outcomes, smell function, polyp recurrence and need for surgery or rescue therapy. Clinical biomarkers predicting response to intranasal LAS, long-term side effects and consequences of discontinuing treatment have been evaluated.

**Methods:**

A retrospective analysis of a database of 60 N-ERD patients seen between 2012 and 2020 was performed in March 2021. They were followed up at 3-months, 1-, 2- and 3-years with upper and lower airway functions assessed at each follow-up.

**Results:**

Higher nasal airflow and smell scores were found at each follow-up in patients taking LAS (*p* < 0.001 and *p* = 0.048 respectively). No influence of LAS on pulmonary function measurements was observed. Patient on intranasal LAS showed a lower rate of revision sinus surgery when compared to those who discontinued the treatment (*p* < 0.001). None of the variables studied was found to influence LAS treatment response.

**Conclusion:**

Our study demonstrates the clinical effectiveness of long-term intranasal LAS in the management of N-ERD in terms of improved nasal airflow and olfaction and a reduced need for revision sinus surgery. Intranasal LAS is safe, being associated with a lower rate of side effects when compared to oral ATAD. However, discontinuation of the treatment at any stage is associated with a loss of clinical benefit.

## Introduction

Non-steroidal anti-inflammatory drug (NSAID)-exacerbated respiratory disease (N-ERD), also referred to as Samter’s triad, remains a diagnostic and therapeutic challenge [1]. Standard treatments include the use of nasal corticosteroids, nasal douches, inhalers, leukotriene-modifying drugs, and biologics targeting type 2 inflammatory cytokines [2]. Endoscopic sinus surgery (ESS) is also used to debulk nasal polyps and improve corticosteroid delivery when CRSwNP is uncontrolled despite optimal medical treatment [2]. Nevertheless, patients with N-ERD tend to undergo up to 10-times more revision ESS and are more likely to be dependent on oral corticosteroids to control their disease [3].

Aspirin treatment after desensitization (ATAD), whereby a patient is exposed to a gradually increasing dose of aspirin until a final daily dose is reached, has emerged as an effective therapeutic option suitable for N-ERD patients with recalcitrant disease [1]. Since its first description in 1980 [4], several blinded and longitudinal studies have consistently shown the benefit of ATAD including a decrease in sinonasal symptoms (Grade 1A), decrease in intranasal corticosteroid use (Grade 2B), reduction in recurrence of nasal polyps (Grade 2B), and decrease in the need for revision surgery (Grade 2B) [1]. According to the American Academy of Allergy, Asthma and Immunology, ATAD is a “unique treatment option that should be considered in all eligible patients with AERD as a means to improve clinical outcomes and delay or prevent future sinus surgery” [5]. The majority of N-ERD sufferers would benefit from ATAD [5]. However, there are some patients who cannot tolerate ATAD because of associated symptoms affecting the skin, gut or lungs. To minimise ATAD-related risks, intranasal administration of lysine-aspirin (LAS) has been suggested as a safer and faster route than oral ATAD [6]. However, the evidence for its use is less strong. Previous trials have demonstrated the beneficial effects of long-term intranasal LAS (75 mg) in the treatment of nasal polyps [7–11] leading to a significantly lower rate of polyp recurrence at 2 years when compared with controls (21% vs 76%) [6]. Nevertheless, a previous small randomized-controlled trial (RCT) on 43 patients with N-ERD treated with a lower alternate-day dose of intranasal LAS (16 mg LAS every 48 h) failed to demonstrate a clinical benefit although it showed a decrease in leukotriene receptors [12].

The long-term effects of ATAD using intranasal LAS (75 mg) remain unknown and we aim to perform a long-term cross-sectional analysis of 60 N-ERD patients on intranasal LAS which follows on from our previous short-term evaluation [9]. LAS long-term efficacy will be evaluated using objective outcomes, smell function assessment, polyp recurrence and the need for rescue medicines and surgery. Our secondary aims are to evaluate potential clinical biomarkers to help predict success and determine which patients would be most likely to benefit from intranasal LAS. In addition, we will evaluate the consequences of discontinuing LAS treatment, the long-term side effects of intranasal LAS and potential pulmonary benefits.

## Materials and methods

### Study design

A retrospective analysis of patients with confirmed or possible N-ERD seen at the Royal National Ear, Nose and Throat Hospital (University College London Hospital, London, UK) between 2012 and 2020 was performed in March 2021. Only those patients who continued the intranasal LAS treatment for a minimum of 3 months were included. This cohort of patients was then followed up at 1, 2 and 3 years. N-ERD patients who stopped intranasal LAS at any point during this time frame but who continued to attend the outpatients’ follow-ups were included to compare their nasal and pulmonary function measurements with those still on intranasal LAS. The study was conducted in accordance with the 1996 Helsinki Declaration and approved by the Research Ethic Committee (Reference 06/Q0301/6).

### Diagnosis of aspirin sensitivity

Patients with nasal polyps with a clear history of respiratory reaction to aspirin and at least one other different Cox-1 inhibitor NSAID were considered aspirin sensitive [13]. In patients with one reaction to aspirin/NSAID or no previous ingestion, diagnosis of N-ERD was confirmed with an intranasal graded aspirin challenge, as previously described [14]. Exclusion criteria to aspirin challenge included pregnancy, a history of an immediate anaphylactic or urticarial reaction to aspirin or NSAID, bleeding diatheses, severe gastro-intestinal disease, patients with grade 3 or larger polyps at the pre-challenge endoscopic examination, or patients considered unable to use such medication regularly [14]. All patients were refractory to standard medical therapy (i.e. long-term nasal corticosteroid drops, regular nasal douches with normal saline and corticosteroid inhalers) and gave written informed consent to LAS nasal challenge and to LAS therapy continuation at home after a positive challenge [i.e. increased symptoms (recorded by a visual analogue scale), plus either 25% or greater decrease in the nasal airway as assessed by acoustic rhinometry (reduction of cross-sectional area) or a 40% decrease in PNIF][14].

### LAS treatment after positive aspirin challenge

Treatment was started at home on the day after the positive challenge using drops (50 μl each) from a freshly prepared 50 mg/ml solution of LAS in sodium chloride 0.9%. The starting dose for therapy was the dose to which the patient had responded intranasally on the previous day plus an extra one drop into each nostril. The patient was given instructions to increase similarly the number of drops each day, up to a maximum of nine drops in each nostril, equivalent to 45 mg of aspirin, until assessment at 3 months (first follow-up). The number of drops was then further increased each day up to a maximum suggested dose of 15–20 drops in each nostril equivalent to 75–100 mg aspirin [9]. This was chosen as it is also an optimal dose for cardiovascular protection [15] and the nasal administration is followed by swallowing the LAS.

### Objective evaluation

At each follow-up upper and lower airway functions were assessed and objective measurements taken were recorded. A portable Youlten peak flow meter (Clement Clark International) was used to obtain the peak nasal inspiratory flow (PNIF), as previously described [16]. The ability to smell was scored using the Nez du Vin system [17], a 6-item suprathreshold identification test (maximum score 6). The lower respiratory function was evaluated using a spirometer (Maids Moreton, UK).

### Data

Population data including demographic, disease onset, number of previous ESS, benefit on anti-leukotrienes and home medications were collected. Skin prick test (grass and tree pollens, house dust mite, cat and dog hair, alternaria, cladosporium, aspergillus) and relevant blood tests (eosinophils count, ANCA positivity, vitamin D3 and aspergillus fumigatus IgG levels) results at baseline were also documented. Details about the aspirin challenge and the dose of intranasal LAS taken at each follow-up were recorded. Objective measurements values, any modification in the patients’ home medications as well as number of courses of oral corticosteroid taken and revision ESS received in between each follow-up were also noted.

### Statistical analysis

Outcome variables are measured repeatedly on the same cohort of individuals at multiple time-points, with the aim to characterize changes in the individuals' measurements over time and their association with clinical factors. A linear fixed effect model [18] has been fitted to the data with random effects on the patients and, if needed, on time. Significant variables were selected by AIC and for the t-tests the Satterthwaite's method [19] was used. Ottaviano et al. [20] showed that the relationship between PNIF and covariates is typically not linear and they proposed a square root transform of PNIF, which has been evaluated appropriate also for our data. The residual analysis of the models suggests that the same transformation is adequate to all the pulmonary variables used here. The Nez du Vin is a discrete quantity thus a generalized linear mixed model with binomial distribution and logic link was fitted. All the analysis has been performed in R (R Core Team, 2021).

## Results

### Database analysis

**Fig. 1:**
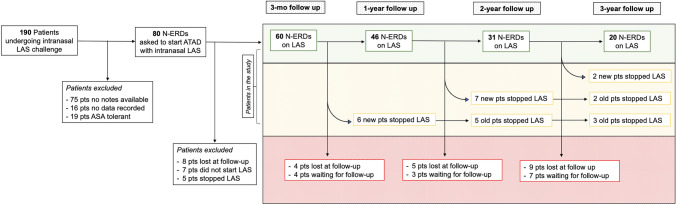
Flow diagram of patients included in the study

Of the 190 patients referred since 2012 with possible N-ERD and who underwent intranasal LAS challenge for diagnosis confirmation, 75 had no notes available for screening at the moment of the analysis while data had not been recorded for 16 patients. A further 19 patients did not show any reaction to the escalating aspirin dose at the challenge and thus were excluded from the study leading to a final population of 80 N-ERD patients who were asked to start on intranasal LAS as part of their treatment. Seven patients never started ATAD with intranasal LAS after the challenge. Sixty patients had at least a 3-month follow-up and they were included in the analysis. Figure 1 shows the flow chart of the study population with the number of N-ERD patients on LAS treatment, those who stopped intranasal LAS and those lost at each follow-up during the study period.

### Population characteristics

The total population was composed of 34 men and 26 women (male to female ratio of 1.3:1) with a median age of 46.5 years. The median age of onset for both rhinitis/chronic rhinosinusitis and asthma was 30 years, followed by nasal polyps (33 years). Before LAS challenge, patients had undergone a median number of 2 revision ESSs and sense of smell was affected in the majority of them (84.6%). The majority of patients (59.6%) had a history of lower airway reaction following aspirin/NSAIDs intake. Detailed characteristics of the population as well as family history of N-ERD and parental ethnicity are reported in Table 1.

**Table 1:** Detailed characteristics of the population

	Subjects (*n* = 60)
**Demographics**	
Age, median [P25–P75], yr	46.5 [39–58.5]
Sex, *n* (%)	
Male	34 (56.7%)
Female	26 (43.3%)
Father ethnicity, *n* (%)	
White	46 (85.2%)
Asian/Asian British	5 (9.3%)
Mixed/Multiple ethnic groups	3 (5.5%)
Mother ethnicity, *n* (%)	
White	44 (84.7%)
Asian/Asian British	5 (9.6%)
Mixed/Multiple ethnic groups	2 (3.8%)
Black Caribbean	1 (1.9%)
Family history, *n* (%)	
Aspirin/NSAIDs sensitivity	4 (7.8%)
Asthma	26 (52.0%)
Rhinitis/rhinosinusitis	20 (40.0%)
Nasal polyps	13 (26.0%)
Rhinitis/CRS onset, median [P25–P75], yr	30 [19.3–39.8]
Nasal polyps’ onset, median [P25–P75], yr	33 [24–40]
Number of polypectomies, median [P25–P75], yr	2 [2–5]
Sense of smell affected, *n* (%)	
Yes	44 (84.6%)
No	8 (15.4%)
Diagnosed asthma, *n* (%)	59 (98.3%)
Asthma onset, median [P25-P75], yr	30 [18.8–40]
History of aspirin/NSAIDs reaction, *n* (%)	
Upper airway	21 (40.4%)
Lower airway	31 (59.6%)
Antileukotrienes benefit, *n* (%)	19 (41.3%)
**Therapy at baseline**	
Long-term nasal corticosteroid drops, *n* (%)	60 (100%)
Nasal douche, *n* (%)	60 (100%)
ICS, *n* (%)	16 (30.2%)
LABA, *n* (%)	1 (1.9%)
ICS + LABA, *n* (%)	33 (62.3%)
SABA, *n* (%)	23 (43.4%)
Anticholinergic inhaler, *n* (%)	2 (3.8%)
Long-term macrolides, *n* (%)	4 (7.5%)
Antileukotrienes, *n* (%)	28 (52.8%)
Oral antihistamines, *n* (%)	25 (47.2%)
**Investigations**	
Skin prick test positivity, *n* (%)	
None	25 (43.1%)
One allergen	9 (15.5%)
2–4 allergens	19 (32.8%)
More than 5 allergens	5 (8.6%)
Aspergillus positivity	4 (6.9%)
ANCA, *n* (%)	
Positive	2 (4.3%)
Negative	45 (95.7%)
Eosinophils, median [P25–P75], × 10^9/L	0.43 [0.26–0.73]
Vitamin D3, median [P25–P75], nmol/L	63 [44–77]
Aspergillus Fumigatus IgG, median [P25–P75], mcg/mL	20.1 [16.9–32.3]
Aspirin challenge, *n* (%)	
Intranasal	50 (83.4%)
Oral	5 (8.3%)
Not performed*	5 (8.3%)
Aspirin dose at challenge, median [P25-P75], mg^+^	
Intranasal	20 [15–47.5]
Oral	100 [100–120]

Valid percentages, not including missing values. Eosinophils reference range: 0.0–0.4 × 10^9/L. Vitamin D3 reference range: 25–120 nmol/L. Aspergillus Fumigatus IgG reference range: 0.00–90.00 mcg/mL

*NSAIDs* non-steroidal anti-inflammatory drugs, *ICS* inhaled corticosteroids, *LABA* long-acting β2 adrenergic receptor agonists, *SABA* short-acting β2 adrenergic receptor agonists

*Not performed because of a clear history of aspirin sensitivity

^+^1 drop ≈ 2.5 mg of lysine aspirin

### LAS treatment drop-out rate and side effects

At 3 months a drop-out rate of 25.0% (20/80) was observed while at the following follow-ups (1, 2 and 3 years) it was, respectively, of 16.7% (10/60), 26.1% (12/46) and 12.9% (4/31). Of those who started LAS but then suspended the treatment over the 3-year follow-up period, 11.3% of patients (9/80) discontinued LAS because of lack of improvement, 3.8% (3/80) for “gut problems”, 2.5% (2/80) because of “worsening of nasal symptoms”, 2.5% (2/80) for an unbearable “nasal burning sensation”, 2.5% (2/80) for the appearance of an urticarial rash, 1.3% (1/80) for the appearance of tinnitus, and 1.3% (1/80) for pregnancy. For those lost to follow-up (26/80) we were not able to record any reasons.

### Investigations at baseline

Skin prick test demonstrated that over half of the patients (56.9%) were atopic, with the majority of them (32.8%) reacting to 2–4 allergens. ANCA was negative in 95.7% of the subjects and the median values for eosinophils, vitamin D and aspergillus fumigatus were within the normal range in the studied population (Table 1). Aspirin challenge was performed in 55 (91.7%) subjects, of which 50 (83.4%) reacted at the nasal challenge stage while 5 (8.3%) required a further oral challenge. Five patients (8.3%) had a clear history of the previous reaction to aspirin or other NSAIDs and the aspirin challenge was not necessary (Table 1).

### Therapy at baseline and during follow-ups

All subjects were on long-term nasal corticosteroid drops (Fluticasone propionate 400 μg), regular nasal douches with normal saline and corticosteroid or corticosteroid plus long-acting beta agonist (LABA) inhalers. Two patients (3.8%) were on anticholinergic inhalers, 4 (7.5%) on long-term macrolides (Clarithromycin 250 mg od), 28 (52.8%) on antileukotrienes (Montelukast 10 mg) with benefit and 25 (47.2%) on oral antihistamines (Table 1). At the follow-ups, no relevant changes to patients’ medical therapy were noted apart from a significant increase of those taking a combination of inhaled corticosteroids and long-acting β2 adrenergic receptor agonists (*p* = 0.021) and a significant decrease of those taking inhaled corticosteroids only (*p* = 0.01).

### Long-term variability of pulmonary and nasal function and effect of LAS treatment

**Fig. 2:**
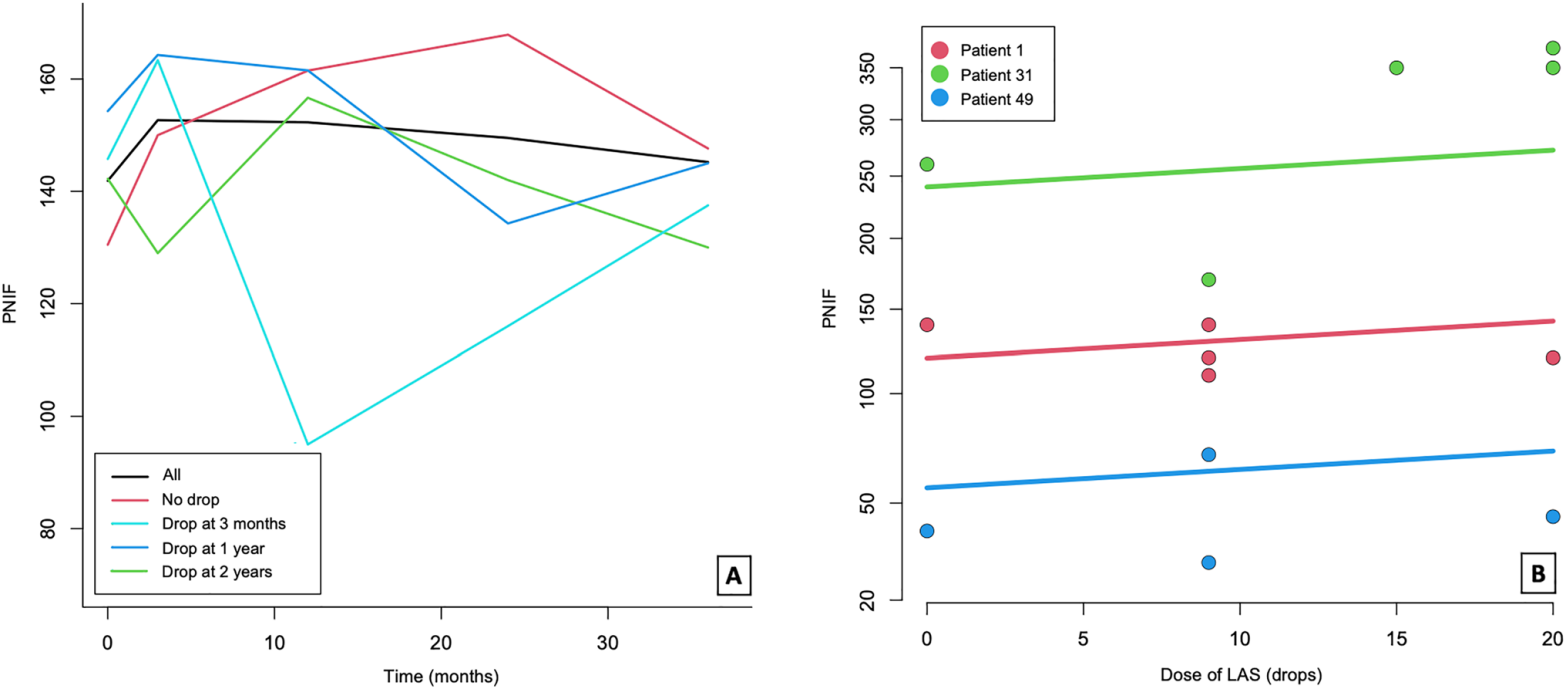
**A** Temporal trend of PNIF mean values in the different groups representing patients who discontinuing the treatment at 3 months, 1 year, 2 years, or not. **B** Relationship between PNIF and the dose of lysine aspirin (LAS) drops in three different patients taken as example

**Fig. 3:**
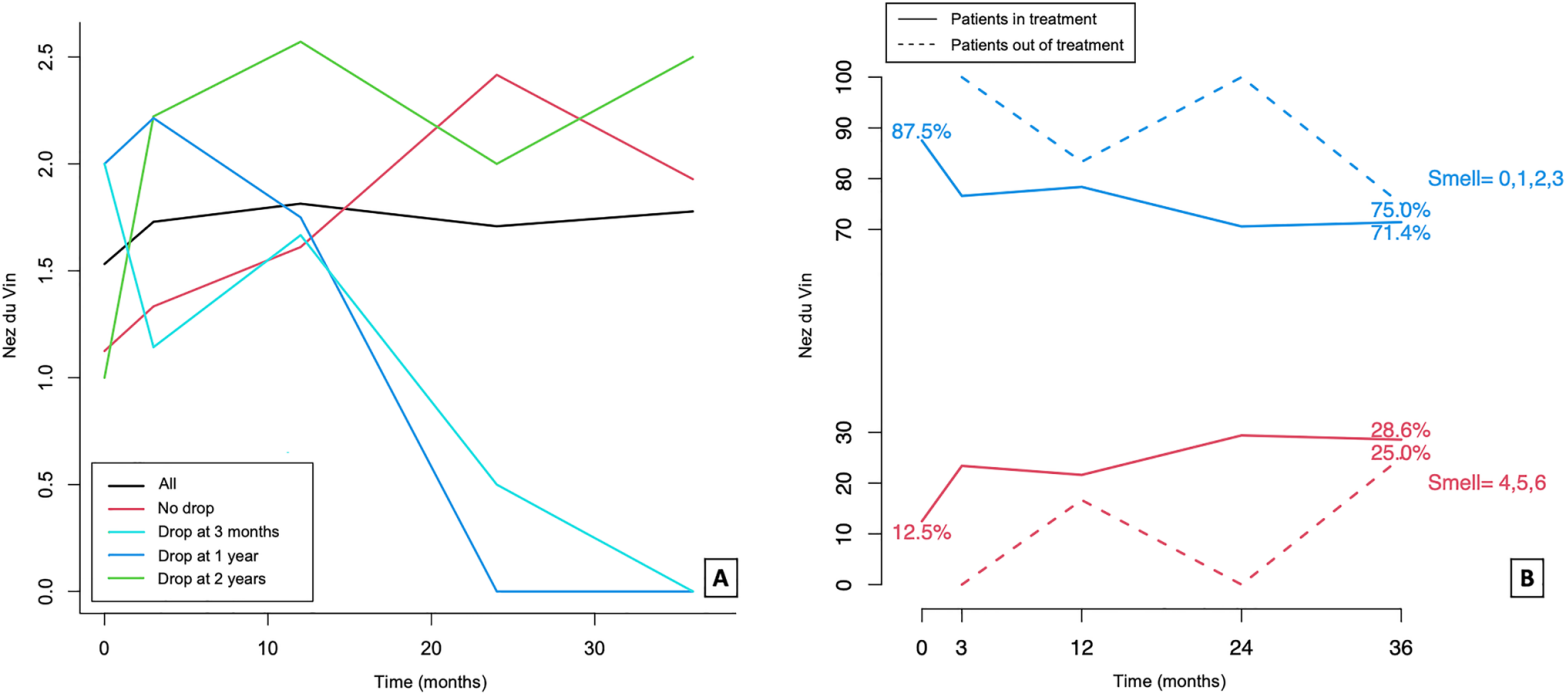
**A** Temporal trend of Nez du Vin mean scores in the different groups representing patients who discontinuing the treatment at 3 months, 1 year, 2 years, or not. **B** Percentage of patients obtaining low (0,1,2,3) and high (4,5,6) scores at Nez du Vin over time according to treatment

**Fig. 4:**
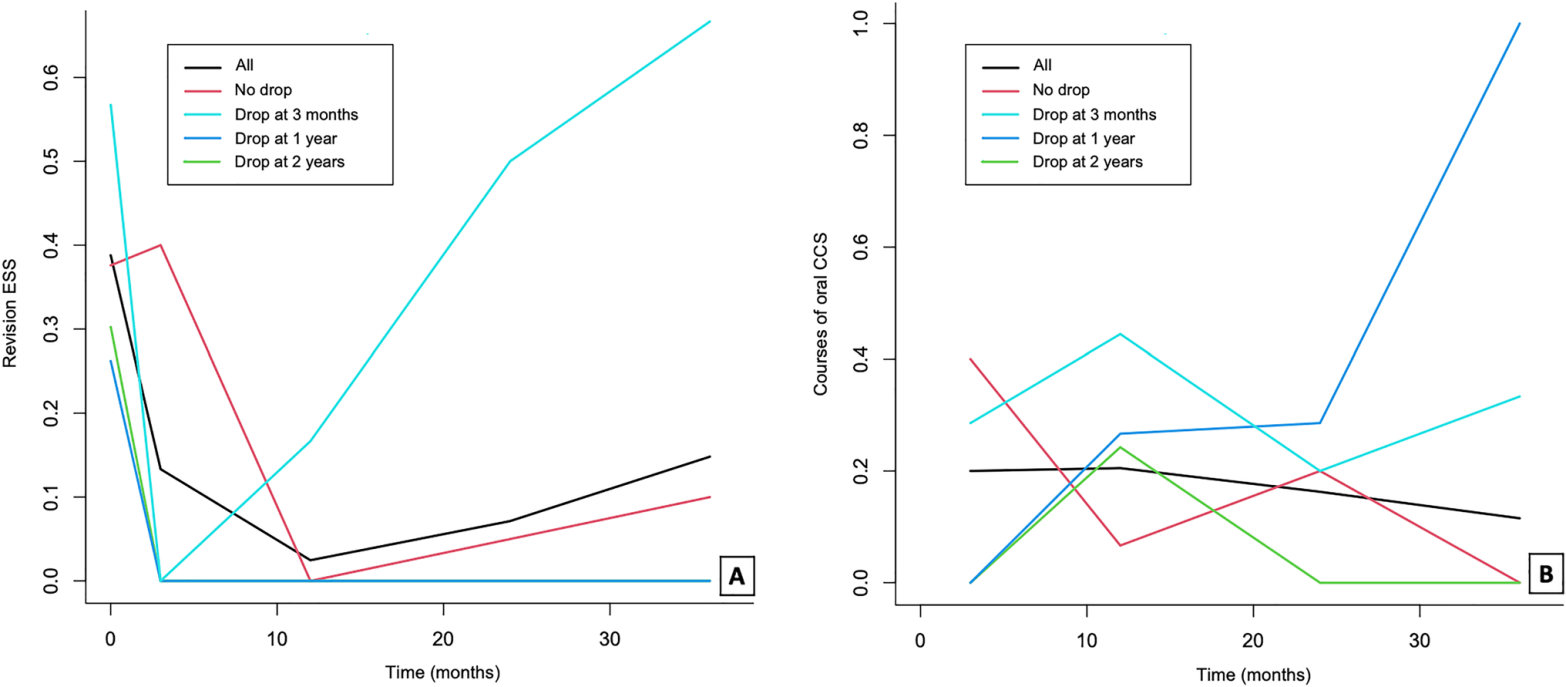
**A** Temporal trend of revision endoscopic sinus surgery (ESS) mean number and (**B**) of the mean number of oral corticosteroid (CCS) courses taken in the different groups representing patients who discontinuing the treatment at 3 months, 1 year, 2 years, or not

PNIF values remained stable during the study period in patients on long-term LAS treatment, but higher values of PNIF and Nez du Vin scores were found at each follow-up in patients taking LAS when compared to those who discontinued it. A significant positive linear correlation between the dose of LAS taken and nasal airflow (average increase of 0.048 at $\sqrt{\mathrm{PNIF}}$ for each drop of LAS taken) as well as the odour identification were demonstrated. No influence of LAS on pulmonary function measurements was observed in patient on LAS nor on those who ceased taking it. An increase in the number of revision ESSs and courses of oral corticosteroid was observed in those who stopped LAS with a significant negative linear correlation found between the dose of LAS taken and the number of revision ESSs. Conversely, no effect of LAS was found on the number of courses of oral corticosteroid taken (Figs. 2, 3, 4; Tables 2, 3).

**Table 2:** Variables at baseline and follow-ups

	Baseline	3-month	1-year		2-year		3-year	
	*n* = 60	On LAS *n* = 60	On LAS *n* = 46	Stopped *n* = 6	On LAS *n* = 31	Stopped *n* = 12	On LAS *n* = 20	Stopped *n* = 7
LAS drops/nostril*	–	10 [9–15]	14 [10–20]	–	15 [10–20]	–	17.5 [11.5–20]	–
PNIF (L/min)	140 [110–170]	150 [120–190]	150 [130–180]	80 [70–100]	150 [130–160]	130 [77.5–160]	140 [107.5–162.5]	115 [102.5–150]
Nez du Vin	0 [0–3]	1 [0–3]	2 [0–3]	0.5 [0–2.5]	2 [0.5–4]	0 [0–0]	1.5 [0.5–4]	0 [0–0]
FEV_1_ (L)	2.7 [2.3–3.6]	2.6 [2.1–3.5]	2.4 [2.1–3.7]	2.9 [2.3–3.4]	2.5 [2.2–3.4]	2.5 [2–3]	2.4 [2–3.7]	2.2 [1.9–2.3]
FVC (L)	3.7 [2.8–4.5]	3.7 [2.7–4.5]	3.4 [2.8–4.6]	3.7 [2.7–4.3]	3.5 [3–4.3]	3.7 [2.5–4.3]	3.6 [2.5–4.7]	2.5 [2.4–3]
FEV_1_/FVC (%)	80.2 [72–85.7]	79.2 [70.7–85.1]	79.6 [73.1–86.2]	80.1 [78.1–93.4]	75.1 [68.4–82.5]	72.2 [68.4–79.3]	81.2 [66.8–87.3]	74.3 [71.6–80.5]
Revision ESS	–	2 (3.3%)	0 (0.0%)	1 (16.7%)	1 (3.2%)	2 (16.7%)	2 (10.0%)	2 (28.6%)
Courses of oral corticosteroids	–	3 (5.0%)	6 (13.0%)	2 (33.3%)	4 (12.9%)	3 (25.0%)	0 (0.0%)	3 (42.9%)

Results shown as median and interquartile ranges for all the variables apart from revision ESS and oral corticosteroids courses where frequencies and percentages were used

*LAS* lysine aspirin, *PNIF* peak nasal inspiratory flow, *FEV*_*1*_ forced expiratory volume in the first second, *FVC* forced vital capacity, *ESS* endoscopic sinus surgery

*1 drop ≈ 2.5 mg of lysine aspirin

**Table 3:** Influence of covariates on selected variables

	Covariates	Partial regression coefficients	*p *value
$\sqrt{\mathrm{PNIF}}$	LAS drops/nostril	0.048	< 0.001
	Age	− 0.036	0.032
	Random effect: patient variance	1.87	
	Random effect: time variance	0.002	0.025
Nez du Vin	LAS drops/nostril	0.029	0.048
	Random effect: patient variance	4.84	
	Random effect: time variance	0.009	< 0.001
$\sqrt{{\mathrm{FEV}}_{1}}$	Sex (male)	0.291	< 0.001
	Age	− 0.013	< 0.001
	Eosinophils	− 0.156	0.026
	Use of inhalers	− 0.312	< 0.001
	Random effect: patient variance	0.001	
	Random effect: time variance	0.0004	0.021
$\sqrt{\mathrm{FVC}}$	Sex (male)	0.373	< 0.001
	Age	− 0.012	< 0.001
	Eosinophils	− 0.151	0.037
	Pre-nasal therapy	− 0.379	0.037
	Nasal therapy	0.171	0.017
	Use of inhalers	− 0.277	< 0.001
	Random effect: patient variance	0.001	
	Random effect: time variance	0.0004	0.039
FEV_1_/FVC	Nasal therapy	< 0.001	< 0.001
	Random effect: patient variance	0.006	< 0.001
Revision ESS	LAS drops/nostril	− 0.014	< 0.001
	Random effect: patient variance	0.139	
	Random effect: time variance	0.0001	0.005

Only significant correlations have been reported where level of significance was greater than *p* < 0.05

*LAS* lysine aspirin, *PNIF* peak nasal inspiratory flow, *FEV*_*1*_ forced expiratory volume in the first second, *FVC* forced vital capacity, *ESS* endoscopic sinus surgery

### Effect of other available variables on nasal and pulmonary functions and on treatment response

A significant negative linear correlation between age and $\sqrt{\mathrm{PNIF}}$, $\sqrt{{\mathrm{FEV}}_{1}}$ and $\sqrt{\mathrm{FVC}}$ (i.e. older age is associated with worse nasal airflow and pulmonary function) was observed while a significant correlation with sex (male) was found only for $\sqrt{{\mathrm{FEV}}_{1}}$ and $\sqrt{\mathrm{FVC}}$ (i.e. male patients have a better pulmonary function). The eosinophil count was found to vary with pulmonary but not nasal function measurements. A significant negative association between the use of inhalers and both $\sqrt{{\mathrm{FEV}}_{1}}$ and $\sqrt{\mathrm{FVC}}$ (i.e. patients taking inhalers have a worse pulmonary function) and a significant positive association between nasal therapy and $\sqrt{\mathrm{FVC}}$ and FEV_1_/FVC (i.e. patients on nasal therapy have a better pulmonary function) were also demonstrated (Table 3).

We did not observe any influence of all the variables studied (sex, age, parental ethnicity, age of disease onset, positivity at skin prick test, eosinophil count, vitamin D3 and aspergillus IgG levels, final aspirin dose at challenge) on the LAS treatment response.

## Discussion

To our knowledge, this study represents the first long-term evaluation of intranasal ATAD demonstrating the long-term (3-year follow-up) effectiveness of intranasal LAS in managing CRSwNP in N-ERD using a dose which is also beneficial to the cardiovascular system [15]. Our results corroborate our previous short-term findings on 105 N-ERD patients where we found a significant increase of PNIF, olfaction and nasal nitric oxide levels following intranasal treatment with LAS at 12-months follow-up [9].

We observed that patients on intranasal LAS showed higher scores of PNIF when compared to those who discontinued the treatment (*p* < 0.001 at the linear fixed effect model) (Tables 2, 3). Moreover, the median PNIF values of those who continued the treatment remained almost stable during the whole length of the follow-up which suggests the efficacy of LAS in controlling polyp growth (Table 2). This reflects previous results obtained with oral ATAD both in the short- and in the long-term despite using a consistently higher dose of daily aspirin (300 mg/day–650 mg twice/day) [21–24], whereas no significant differences in endoscopic polyp scores have been reported by an RCT in patients on low-dose oral aspirin (100 mg/day) [25].

Sense of smell is more impaired in patients with N-ERD when compared to the aspirin-tolerant counterpart [13, 26] and odour identification has been found to be the most affected ability as measured by means of a validated reliable olfactory test [26]. In our population we observed a significant improvement in smell function (i.e. identification) in patients on long-term intranasal LAS (*p* = 0.048 at the linear fixed effect model) when compared to those who stopped the treatment. (Tables 2, 3; Fig. 3B) Similar findings have been reported in patients on high-dose of oral aspirin (300 mg/day [26] or 650 mg twice/day [27]), but once more, no significant difference in olfaction were observed when using a lower daily oral aspirin dose (100 mg/day) [25]. Nevertheless, a meta-analysis, which considered also 5 RCTs, did not demonstrate significant changes in smell scores in N-ERD patients receiving a high maintenance dose of oral aspirin (650 mg/day) [28].

We also observed a significantly lower rate of revision ESS in patients on long-term intranasal LAS when compared to those who discontinued the treatment (*p* < 0.001 at the linear fixed effect model) (Tables 2, 3) which indirectly confirms the ability of LAS to reduce nasal polyp’ growth and recurrence rate. In particular, the median number of operations per year changed from 0.24 (roughly one operation per 4 years) to zero operation in the 3-year follow-up period. Moreover, even if the rate of revision ESS for N-ERDs on LAS increased in the last two years of follow-up this remained considerably lower than that of those who discontinued the treatment (Tables 1, 2, 3). This mimics previous findings by Stevenson et al. in a long-term follow-up study of 65 ASA-sensitive treated with oral ATAD who found a concomitant decline of sinonasal surgery from one operation per 3 years to one operation per 9 years [24].

Even though the percentage of oral corticosteroid courses taken was higher at each follow-up in the group of N-ERD patients who suspended the treatment (Table 2), this difference was not statistically significant. However, a significant reduction in annual oral corticosteroid requirements was found with oral ATAD (daily dose of aspirin between 325 and 650 mg) [29].

We did not observe a significant change in pulmonary function between those on long-term intranasal LAS and those who stopped LAS treatment; however, we did demonstrate that spirometry measurements remained stable over time in our LAS population (Table 2). Even if these results suggest an inability of intranasal LAS to improve breathing, we did demonstrate that LAS intake does not adversely affect lung function in the long-term. Conversely, oral ATAD has been shown its efficacy to be strongest in improving asthma symptoms and pulmonary outcomes [28–31]. This incapacity of intranasal LAS to improve pulmonary function may explain the lack of a significant reduction in the number of oral corticosteroid courses taken by those on long-term treatment where this is needed to control asthma exacerbations.

Patient concordance with such a long-term treatment requires continued clinician input and monitoring and strong patient motivation. A higher drop-out rate is expected to happen in the first months because of the initial side effects, poor compliance with the complicated treatment and/or the lack of symptomatic improvement. In our retrospective study, the drop-out rate was of 37.5% at 1 year but this went down in the following 2 years of follow-ups (respectively, 26.1% and 12.9%). The majority of N-ERD patients who discontinued intranasal LAS did so because of an absence of improvement (11.3%). However, a lack of clinical benefit has also been reported to be a common reason for treatment suspension in oral ATAD [29]. To differentiate potential responders from non-responders to aspirin treatment, many researchers have tried to identify biomarkers able to predict a positive response to ATAD. Female sex, high blood eosinophil count, low sputum neutrophil percentage, severe nasal symptoms, high hydroxyprostaglandin dehydrogenate, and low proteoglycan 2 gene expression have recently been shown to be good predictors for a positive response to oral ATAD (650 mg/day) [32]. Patients with an inflammatory neutrophilic phenotype are unlikely to respond to aspirin treatment [32] and a recent study found that the use of anti-leukotrienes reduces the response to LAS nasal challenge [33]. However, we were not able to find any correlation between the variables studied and intranasal LAS treatment response failing to confirm our previous findings of higher PNIF and smell scores in allergic patients and those with later N-ERD onset [9].

Daily oral administration of high-dose of aspirin represents the gold standard for ATAD [5] but it is affected by a high incidence of side effects (8–46%) [6]. These include naso-ocular reactions (90%), bronchial/laryngeal (43%) or gut (23%) problems and skin reactions (10%) [34]. Intranasal administration of LAS is better tolerated and has a lower rate of side effects when compared to oral aspirin [9]. In our current study, only 3.8% of the patients on LAS complained of gut problems, while 2.5% reported a worsening in their nasal symptoms, nasal burning sensation (2.5%) or had an urticarial rash (2.5%). The same rate of gut problems (3.8%) was found in our previous audit on N-ERD patients treated with intranasal LAS, confirming the lower risk of gastrointestinal side-effects linked to intranasal aspirin administration [9].

A consensus does not exist on the exact daily dose of aspirin which should be offered. Nucera et al. used significantly lower doses of intranasal LAS (initial dose of 20 µg progressively increased to a maintenance dose of 4 mg six times/week) than ours and observed a favourable effect of LAS in nasal polyposis [7]. Sousa et al. showed that doses of 16 mg of intranasal LAS daily reduced leukotriene receptors but had no clinical effect [35]. Ogata et al. using 30 mg of intranasal LAS daily did find clinical benefit on PNIF [36]. In our study, the median dose of daily intranasal LAS ranged from 50 mg at 3 months to 87.5 mg at 3 years whereas, according to the EAACI position paper, a dose of 75 mg/day “may be effective to relieve symptoms of CRS” [1]. A comparable variety in the maintenance oral dose of aspirin has been reported in a recent meta-analysis where this ranged between 100 and 1300 mg daily [28]. For intranasal LAS, we recommend to reach a final dose of 15 drops of LAS/nostril/day which corresponds to a total of 75 mg of LAS/daily. This represents also an ideal cardiovascular protective dose [15]. However, in our experience some patients may benefit from a higher dose of LAS, with some of them taking up to 150 mg of LAS/daily without any nasal discomfort. This is supported by the fact that a linear positive correlation between $\sqrt{\mathrm{PNIF}}$ and the dose of LAS was demonstrated in our study. (Fig. 2B).

Since nasal polyps represent an obstacle to intranasal LAS activity, a role for ASA desensitization therapy following recovery from ESS has been advocated in the management of N-ERD. [37] Even though we do not routinely perform ESS before starting the intranasal LAS, for the reason above-mentioned patients with a polyp grade of 3 or 4 at the endoscopic examination performed before the LAS challenge are considered ineligible to start LAS desensitization. Conversely, they are treated, either medically with oral and intranasal corticosteroids, or surgically, to reduce polyp size prior to the challenge. [14]

So far, it is not known, once ATAD is started, when it could be suspended without losing the beneficial effects gained. To our knowledge, this is the first study to have demonstrated that the suspension of intranasal LAS treatment at any point of the follow-up period is associated with a worsening of the nasal airflow and olfaction as well as an increase in the need for revision ESS. Therefore, we recommended to attempt an initial trial period of 3 months on LAS to determine whether the patient notes a clinical improvement. For patients who respond to treatment, we suggest a continuation of intranasal LAS indefinitely.

### Study strengths and limitations

The present study has the longest follow-up for intranasal LAS desensitisation with a dose which is beneficial to the cardiovascular system [15]. However, the retrospective design of our study constitutes a limitation due to the intrinsic limit of data homogeneity and availability in retrospective studies. For instance, we were unable to retrieve patient-reported outcomes measures (PROMs) which represents a lack in our data collection. Even if the drop-out of patients at each follow-up is a further limitation, on the other hand it allowed us to evaluate what happens when a N-ERD patient discontinues intranasal LAS treatment.

## Conclusion

Our study demonstrates the long-term clinical effectiveness of intranasal LAS in the treatment of N-ERD in terms of improved nasal airflow, olfaction and a reduced need for rescue surgery. However, treatment discontinuation at any stage is associated with a loss of clinical benefit. Additionally, intranasal LAS is associated with a lower rate of side effects when compared to oral ATAD. New biologics may provide substantial benefit to patients with N‐ERD but represent an expensive option. Intranasal LAS can be a highly cost-effective and safe treatment option when compared to revision ESS or biologics and should be offered, if possible, before other treatments are considered.

## Data Availability

Not applicable.
